# A nationwide approach to reduction in anaesthetic gas use: the Dutch Approach to decarbonising anaesthesia

**DOI:** 10.1016/j.bja.2024.11.049

**Published:** 2025-01-30

**Authors:** Jasper M. Kampman, Egid M. van Bree, Lieke Gielen, Nicolaas H. Sperna Weiland

**Affiliations:** 1 Department of Anaesthesiology, Amsterdam UMC, University of Amsterdam, Amsterdam, the Netherlands; 2 Centre for Sustainable Healthcare, Amsterdam UMC, Amsterdam, the Netherlands; 3 Department of Surgery, Maastricht University, Maastricht, the Netherlands; 4 Dutch Society for Anaesthesiology, Utrecht, the Netherlands

**Keywords:** anaesthetic gas, carbon footprint, environmental impact, greenhouse gas, inhalation anaesthesia, professional standard, sustainability, volatile anaesthetic

## Abstract

Anaesthetic gases account for ∼3% of the carbon footprint of the entire healthcare sector and up to 63% of the emissions originating from surgical care. Transitioning to predominant use of total intravenous anaesthesia (TIVA) has been proven a safe and effective strategy to reduce this footprint, yet its adoption has been slow in most countries. Interventions at the national level have been limited to regulatory action (e.g. banning of desflurane) and publication of nonbinding recommendations and best practices. We describe a new approach that we used to drive sustainable change and apply it to the debate between TIVA and inhalation anaesthesia at the national level. The Dutch Approach is founded on a bottom-up, self-regulatory model grounded in evidence-based practices. Patient safety studies, a national inventory of anaesthetic drug use, and in-depth interviews with anaesthetists were combined in developing a national guideline. Meeting the two main concerns among anaesthetists, patient safety and professional autonomy, the guideline requires all Dutch anaesthetic practices to adopt a local protocol whose main message is ‘TIVA when possible, inhalation anaesthesia when necessary’. Central to the approach was the integration within the national quinquennial quality control audits. Adoption and implementation will be monitored and evaluated in an ongoing research project.

**Editor's key points**

• Anaesthetic gases are a major contributor to the carbon footprint of surgical care, and total intravenous anaesthesia (TIVA) is a safe and effective strategy to reduce this footprint.
• This paper describes a national, bottom-up, self-regulatory and evidence-based approach to drive sustainable change in reducing carbon emissions from the use of anaesthetic gases.
• This ‘Dutch Approach' included patient safety studies, a national inventory of anaesthetic drug use, and in-depth interviews with anaesthetists that were used to develop a national guideline that requires all Dutch anaesthetic practices to adopt a local protocol to use ‘TIVA when possible, inhalation anaesthesia when necessary’.
• Adoption and implementation will be monitored by mandatory quality control audits and evaluated in an ongoing research project.

In high-income countries, the healthcare sector represents a substantial part of the national economy. In the Netherlands, the sector employs 14% of the working population with an annual budget of more than €100 billion (13% of Dutch GDP). It has been estimated that healthcare-related environmental impact is responsible for 7.3% of the Dutch carbon footprint and 13% of its material extraction (mainly minerals and metals extracted abroad).^1^ Within the healthcare sector, surgical care is a major contributor to greenhouse gas emissions.^2^ When inhalation anaesthesia is used, up to 63% of the carbon footprint of surgical care originates from the delivery of anaesthesia^2^ and atmospheric emissions of anaesthetic gases account for roughly 3% of the footprint of the entire healthcare sector.^3^

There is ample evidence that using total intravenous anaesthesia (TIVA) instead of inhalation anaesthesia can reduce healthcare-related environmental pollution.^4^^,^^5^ In recent years, several tactics have been used to drive this sustainable change on a national level. In some countries, a reduction of anaesthetic gas use is enforced through rules and regulations, such as the 2023 ban on desflurane use in Scotland.^6^ Others encourage change through the publication of nonbinding guidelines, such as the consensus paper by the European Society of Anaesthesiology^7^ or the recommendations by the Belgian Society of Anaesthesiology.^8^ Meanwhile, procurement data from nine European countries (including UK, Italy, and Portugal) revealed that use of anaesthetic gases remained constant overall.^9^ A recent systematic review that studied interventions on sustainability in the operating theatre found that none had a sustained effect on environmental impact, and concluded that very few studies explored barriers to change.^10^

To tackle these prevailing problems and create durable change, we have developed a novel approach. We pioneered the approach by applying it to the debate between TIVA and inhalation anaesthesia. The project was initiated and directed by the Dutch Society for Anaesthesiology (the NVA) and funded through a subsidy provided by the Dutch Ministry of Health, Welfare and Sport. The NVA is a member organisation responsible for guideline development and quality control of all Dutch anaesthetic practices. In the decentralised Dutch healthcare system, each medical profession is represented by its own member organisation which democratically establishes current professional standards that physicians need to adhere to. Although membership is not mandatory, most Dutch anaesthetists (in training) are, with a member file of about 2500. The highest body within the NVA is the members council, consisting of anaesthetists who are chosen by NVA members as their representatives. The anaesthetists on the board of the NVA are chosen by the members council. Employees of the NVA have a supportive function and have no involvement with the clinical content of guidelines or other documents.

The current project culminated in the development of a national guideline on the sustainable use of anaesthetic drugs. These guidelines are self-regulatory and bottom-up in nature, and are used by the NVA in the quinquennial quality control audits. These audits are a mandatory part of the license to operate an anaesthetic practice in the Netherlands. To acknowledge the nationwide nature of this initiative, we have called it the ‘Dutch Approach’. In this article, we describe this novel approach and detail how it was applied to the debate between TIVA and inhalation anaesthesia.

## The Dutch Approach

**Fig 1:**
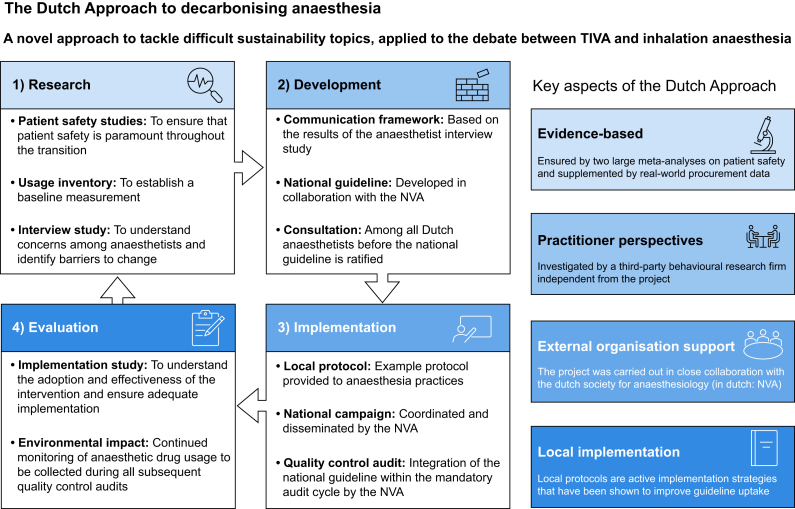
The Dutch Approach, applied to the debate between the use of TIVA and inhalation anaesthesia. TIVA, total intravenous anaesthesia.

The overall goal of the Dutch Approach is to reach a consensus that is acceptable to and supported by all sides of the debate. It is designed to build upon a solid evidence base, consider the perspectives and concerns among practitioners, and create change that is nationwide and embedded in clinical practice. It consists of the following phases (Fig. 1). (1) **The research phase**: all relevant aspects of the debate are investigated. For the issue of TIVA *vs* inhalation anaesthesia, this phase covered a national inventory of anaesthetic drug usage, two systematic reviews and meta-analyses on patient safety, and an interview study with anaesthetists to identify barriers and facilitators to change. (2) **The development phase**: in this phase, the intervention is designed. The national guideline was developed and reviewed by all NVA members. Additionally, a communication framework was developed considering the concerns and barriers identified in the interview study. (3) **The implementation phase**: a nationwide campaign was developed to promote the new guideline and mandatory local protocol. Adherence will be examined in the subsequent quinquennial quality control audits that are mandatory for all anaesthetic practices in the Netherlands. (4) **The evaluation phase**: the reach and adoption of the national guideline are assessed and possibilities for improvement identified. Continued monitoring of anaesthetic drug usage is collected during the quality control audits.

### Phase 1: research

#### Patient safety

Patient safety is paramount throughout the transition towards a more sustainable anaesthesia practice. To ensure that patient safety was not affected by reducing inhalation anaesthesia, we performed two comprehensive systematic reviews and meta-analyses. The first compared general anaesthesia using TIVA *vs* general anaesthesia using inhalation anaesthesia. The second examined the influence of nitrous oxide added to general anaesthesia *vs* general anaesthesia without nitrous oxide. Both systematic reviews included RCTs published from inception to August 2023 and covered endpoints from the core and extended outcome set that was recently published in a series of consensus statements by the Standardised Endpoints in Perioperative care (StEP) expert groups.^11^

In short, the meta-analysis comparing TIVA with inhalation anaesthesia (317 RCTs, 51 107 patients) concluded that postoperative mortality and organ-related morbidity were similar in both groups. TIVA offered advantages for multiple recovery metrics, including postoperative nausea and vomiting (PONV), emergence delirium, and quality of recovery (QoR-40) score.^12^ The meta-analysis for nitrous oxide (71 RCTs, 22 147 patients) concluded that addition of nitrous oxide to general anaesthesia did not influence postoperative mortality and organ-related morbidity.^13^ Nitrous oxide increased the incidences of atelectasis and PONV, whereas it decreased the intraoperative opioid use and time to extubation. The overall findings established that patient safety is not affected by omitting anaesthetic gases.

#### Anaesthetic drug usage

The 135 anaesthetic practices in the Netherlands were contacted between March 2023 and September 2023 to distribute a questionnaire on anaesthetic drug usage in the years 2019–22 (Supplementary Appendix 1). The usage data from all Dutch academic hospitals could be extracted from the hospital pharmacy procurement system (ZAGIS) for the years 2017–23. Participating practices were representative for hospitals in the Netherlands with a total response rate of 57%: seven academic hospitals (100%), 16 major teaching hospitals (62%), 25 regular hospitals (45%), 27 private clinics (61%), and two others (66%). The 77 participating anaesthetic practices employed a total of 900 anaesthetists (full-time equivalents), and performed 600 000 anaesthesia cases and more than 100 000 procedural sedations annually.

Hospital pharmacy procurement figures were collected for sevoflurane, desflurane, isoflurane, and propofol. Not all institutions provided data on every anaesthetic drug in the survey. For sevoflurane, total volume decreased from 7051 L in 2019–4687 L in 2022, constituting a 34% reduction. The total volume of desflurane decreased by 92% from 193 L in 2019 to 15 L in 2022, and that of isoflurane decreased by 71% from 235 L in 2019 to 68 L in 2022. Total volume of propofol, measured in kilogram of the active pharmaceutical ingredient, increased by 28% from 459 kg in 2019 to 586 kg in 2022.

**Fig 2:**
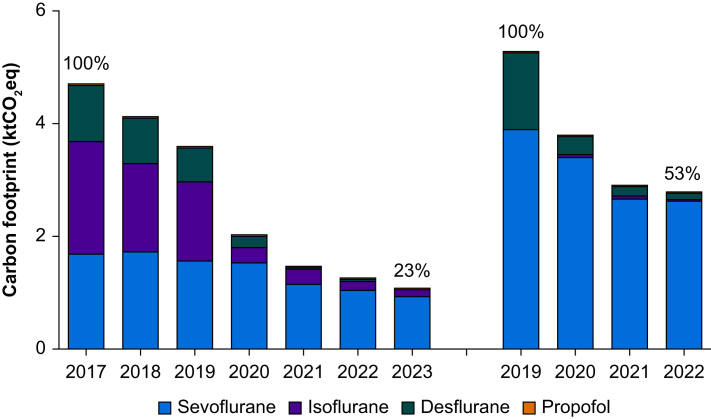
Carbon footprint associated with anaesthetic drug usage in kilotonne (1000 metric tonnes) of CO_2_-equivalents for all Dutch academic hospitals (on the left; 2017–23; 77% reduction; *n*=7) and approximately half of nonacademic hospitals (on the right; 2019–22; 47% reduction; *n*=70).

The carbon footprint was calculated for all anaesthetics (Box 1 and Table 1). The calculation uses the global warming potential (GWP_20_), which is the relative impact of different greenhouse gases over 20 yr in comparison with CO_2_ which has, by definition, a GWP of 1. Inhaled anaesthetics have a relatively short atmospheric lifetime (1–14 yr), so GWP_20_ is the most appropriate time period.^20^ The total carbon footprint of the included anaesthetic drugs was reduced by 61% from 9.97 kt (kilotonnes; 1000 metric tonnes) in 2019 to 3.87 kt in 2022 (Fig. 2). The carbon footprint attributed to propofol over the same period increased from 0.018 kt to 0.026 kt, representing 0.7% of the total carbon footprint of the four anaesthetic drugs in the last available year. A kilotonne of CO_2_-eq is 1 000 000 kg of CO_2_-eq, which is equivalent to roughly 10 000 trips by air from Amsterdam to Paris (source: ecopassenger.org ^21^).

Box 1 **Carbon footprint calculation of anaesthetic drugs.**

**Formula for waste gas emissions:** CO_2_-eq (kg) = density (g ml^-1^) × volume (L) × GWP_20_

**Parameters:** For sevoflurane, desflurane, isoflurane, and nitrous oxide, the respective GWP_20_ are 505, 6930, and 1920.^14^ Respective densities (at room temperature) are 1.505, 1.465, and 1.502.^15^, ^16^, ^17^ Agent manufacturing produces 26, 57, and 50 kg of CO_2_-eq per litre of gas, respectively.^18^^,^^19^

**Propofol:** Published data on its full life cycle (cradle to grave) indicates that 40 kg of CO_2_-eq are emitted for every 1 kg of active pharmaceutical ingredient of propofol consumed.^18^

Alt-text: Box 1

**Table 1:** Anaesthetic drug usage volumes and associated carbon footprint in kilotonnes (1000 metric tonnes) of CO_2_-equivalents for all Dutch academic hospitals (*n*=7) and roughly half of nonacademic hospitals (*n*=70).

	Academic hospitals							Nonacademic hospitals			
	2017	2018	2019	2020	2021	2022	2023	2019	2020	2021	2022
	*Volume in litre (gas) or kilogram active pharmaceutical ingredient (propofol)*										
Sevoflurane	2226	2286	2074	2022	1525	1390	1237	5137	4480	3518	3458
Desflurane	98	80	59	19	1	4	0	134	30	16	11
Isoflurane	693	541	485	96	95	53	43	0	21	18	12
Propofol	208	217	235	282	359	326	320	258	332	384	316
	*Carbon footprint in CO*_*2*_*-eq*										
Sevoflurane	1.692	1.737	1.576	1.537	1.159	1.056	0.940	3.904	3.405	2.674	2.628
Desflurane	0.995	0.812	0.599	0.193	0.010	0.041	0.000	1.360	0.305	0.162	0.112
Isoflurane	1.999	1.560	1.399	0.277	0.274	0.153	0.124	0	0.061	0.052	0.035
Propofol	0.008	0.009	0.009	0.011	0.014	0.013	0.013	0.010	0.013	0.015	0.013
Total CO_2_-eq	4.694	4.118	3.583	2.018	1.457	1.263	1.077	5.274	3.784	2.903	2.788

#### Understanding anaesthetists

To enhance understanding of the barriers to sustainable change among anaesthetists, we performed interviews to collect their concerns and ideas (interview guide in Supplementary Appendix 2). The semi-structured in-depth interviews were performed by two experienced researchers from an independent behavioural research company (SUE & The Alchemists, Amsterdam, the Netherlands) to safeguard privacy and independence from the project. Based on the open questions that were included in the usage inventory questionnaire, subjects with strong opinions, pro and con, were recruited for the interviews. A total of eight anaesthetists were included through purposive sampling to reach a representative group in terms of gender, geographic location, type of practice, experience, and views on anaesthetic gas use. The main finding was the importance anaesthetists place on professional freedom in their practice. Another important finding was that anaesthetists see patient safety as their number one priority. They are proud of their perioperative expertise and passionate about innovation and further enhancing the quality of their daily practice, which includes improving environmental sustainability.

Anxieties that were associated with the switch from inhalation anaesthesia to TIVA included worries about control over anaesthetic depth, the loss of end-tidal concentration monitoring, longer time to awakening when too much propofol is administered, and loss of the comfort that comes with many years of experience with anaesthetic gases. This was offset by recognised gains of TIVA, which included less nausea and agitation after waking up and the notion of working in a more environmentally friendly way. Those with more TIVA experience found monitoring of anaesthetic depth comparable with inhalation anaesthesia.

### Phase 2: development

The NVA, the Dutch professional association for all anaesthetists is tasked with overseeing and safeguarding quality-related matters. It oversees the development of guidelines related to anaesthesia, which range from preoperative screening to the organisation of the PACU. The guidelines detail mandatory practices for anaesthesia departments in the Netherlands in order to mitigate unwanted practice variations and drive advances in quality of care. Guidelines are not directly enforced by the NVA, but can become admissible in two ways. The first is their use by the health inspection and by the disciplinary courts, which use the NVA guidelines to establish common and accepted anaesthetic practices. The second is during the quality audits, performed by an *ad hoc* committee of anaesthetists appointed by the NVA. This committee puts all local protocols through a collegial test to see whether its contents make clinical sense and follow the spirit of the guidelines. Every practice undergoes a quality audit once every 5 yr. In case a department fails to adhere to the national guidelines, a conditional report is issued which obliges the department to initiate the desired change. This is closely monitored until the appropriate practices are implemented.

To integrate sustainability in clinical practice nationwide, the NVA guideline that describes the perioperative process was supplemented with a novel module on the sustainable use of anaesthetics (Supplementary Appendix 3). The insights from the interview study were used to create a communication framework used in the design and dissemination of the guideline. Patient safety aspects were put at the core of the framework along with the professional autonomy among anaesthetists to use their own judgement in this regard. The key message was formulated as ‘TIVA when possible, inhalation anaesthesia when necessary’. The guideline module was drafted by the authors of this manuscript and appointed by the democratically elected board of the NVA. This working party was further supported by the NVA committee on science and innovation, the professional standard committee, and the task force on sustainability. These groups consist of NVA members committed to the subject and appointed by the NVA board.

## Phase 3: implementation

The national guideline underwent a consultation phase during which all Dutch anaesthetists could provide comments. More than 100 comments were submitted which illustrated the polarised nature of the debate. Comments calling for stricter policies on sustainability were contradicted by comments advocating that sustainability should never be a factor in clinical practice. The overall consensus in the working party was that the proposed guideline was properly nuanced between comments from both sides. Based on commenter suggestions, information was added on mitigating the environmental impact of anaesthesia beyond anaesthetic gases. The modified version was ratified by the board and the members council of the NVA. The guideline obliges departments to adopt a local protocol on sustainable use of anaesthetic drugs. This local protocol should confirm that TIVA is the default option for providing general anaesthesia. Departments can list indications for inhalation anaesthesia, allowing individual anaesthetic practices to adapt the local protocol to their own preferences and preserve their professional freedom. Departments can draft their own protocol, but are also provided with an example protocol from the NVA (translated example protocol in Supplementary Appendix 4).

Following ratification of the new national guideline in May 2024, subsequent quality audits will evaluate the presence and adherence to the local protocol on inhalation anaesthesia. During preparation of the audit, departments are requested to provide information to the auditors. Going forward, this will include anaesthetic procurement data. These data serve to identify front-runners and laggards, and to evaluate the environmental impact against the 2019–22 inventory.

## Phase 4: evaluation

In order to understand adoption, implementation, and effectiveness of the intervention, an implementation study will be conducted based on the Consolidated Framework for Implementation Research (CFIR) Outcomes Addendum.^22^ A national survey, building on the earlier anaesthetic drug usage survey, will be used to gather data on the local adoption and implementation of the national guideline. The corresponding local protocol, the communication and dissemination strategy, and the quality audits will be included in the evaluation. To gain additional understanding of determinants that influence implementation, constructs of the Normalisation Process Theory (NPT)^23^ will be studied through inclusion of items of the validated Dutch version of the Normalisation Measurement Development (NoMAD) questionnaire.^24^ If necessary, further qualitative assessments will be used to gain more in-depth understanding based on purposive sampling. These will be small scale targeted studies aimed at understanding daily choices and behaviour of anaesthesia providers. Findings are expected to aid in monitoring and maintaining the evaluation and improvement of the national guideline.

## Discussion

We describe the use of a new method to drive sustainable change on a national level, building upon a solid research phase and without unduly restricting practitioners' autonomy. We pioneered this ‘Dutch Approach’ by applying it to the debate between TIVA and inhalation anaesthesia.

We established a robust evidence-base regarding patient safety, collected anaesthetists' thoughts and concerns regarding TIVA use, and created a national guideline requiring Dutch anaesthetic practices to adopt a local protocol on sustainable use of anaesthetic agents. The key message was formulated as ‘TIVA when possible, inhalation anaesthesia when necessary’. Adherence is ascertained by integration of the guideline within mandatory quinquennial quality audits. Specific attention was paid to the values and concerns among anaesthetists to avoid barriers to implementation.

In most European countries, anaesthetic gas use was stable over recent years.^9^ An explanation may be that current nationwide initiatives to reduce their use were limited to regulatory action^6^ or nonbinding recommendations.^7^ Several alternative interventions have shown effectiveness in reducing the use of anaesthetic gases, including raising awareness,^25^ improving TIVA skills,^26^ and implementing local protocols.^5^^,^^27^ However, these studies only included one or a few hospitals and measured only short-term effects.

A recent systematic review on the implementation of environmental interventions in the operating theatre found that no publications exist on fully scaled implementation projects, and that there is no evidence for a sustained effect of any of the interventions.^10^ This suggests a gap between the evidence behind these interventions and the actual uptake in clinical practice. A recent systematic review on local implementation of centrally driven guidelines revealed several factors of importance, including: perception of guidelines as evidence-based and relevant, involvement of an external organisation to add validity and weight to the policies, and requesting local involvement in the form of a protocol.^28^ Our approach uses these recommendations, including the collaboration with the National Society for Anaesthesiology (NKA), which we believe is crucial for success. It helped us reach as many anaesthetists as possible in the research phase and was essential in disseminating and implementing the resulting national guideline. Integration within the nationwide quality audits will ensure that all anaesthesia practices incorporate sustainability into their local protocols. The ongoing study to evaluate the approach should help prevent a ‘type III error’: no effect of the intervention owing to inadequate implementation.^29^ Ultimately, promoting sustainable use of anaesthetic gases might lead to additional change in other behaviours related to sustainability in operating theatres (e.g. recycling or powering down equipment after use). We hypothesise that our approach will also be effective for other difficult transitions that the healthcare sector faces in the transition towards a sustainable industry, especially when it concerns a polarised issue.

The results of the usage inventory might reflect a situation unique to the Netherlands and underestimate the potential for reducing the carbon footprint of anaesthetic care worldwide. Firstly, only half of the anaesthetic practices in the Netherlands provided procurement figures. Secondly, 98% of the volume of inhaled anaesthetics in our data was sevoflurane, while in the USA and Canada about half of the administered volume was desflurane.^25^^,^^30^ A worldwide 2023 survey found that 10–20% of anaesthetists use desflurane.^31^ Considering that desflurane is administered at a several-fold higher concentration than sevoflurane, roughly half of the total worldwide volume of inhaled anaesthetics might be desflurane. The GWP of desflurane is over more than 10-fold higher than that of sevoflurane, which means that desflurane was by far the most impactful volatile anaesthetic worldwide.^32^ This is contrary to our data reflecting the Dutch landscape of inhaled anaesthetics where sevoflurane accounts for the majority of the carbon footprint. Thirdly, our inventory revealed that general anaesthesia was usually maintained using TIVA. This is in stark contrast with the global trend in which roughly 80% of general anaesthesia is maintained using inhalation anaesthesia.^31^^,^^33^^,^^34^ Fourthly, nitrous oxide use is low in Dutch practice. In our usage inventory, nitrous oxide was ultimately omitted from the final report because several hospitals with piped manifolds did not report usage data, or only cylinder use, after which we considered the data incomplete. However, previous publications have reported low use of nitrous oxide, both in the operating theatre and the maternity unit.^35^ These limitations mean that the results of our inventory represent a major underestimation of the potential carbon savings of adopting a more sustainable use of anaesthetic gases.

When inhalation anaesthesia is used, it can be the largest source of carbon emissions originating from operating theatres.^2^ However, it is certainly not the only source of healthcare-related emissions. The current project focussed exclusively on inhalation anaesthesia because we wanted to avoid developing a document with mere nonbinding recommendations, but instead design a national guideline that could build on a robust evidence base, was unambiguous, and expressly usable for audits. Other important aspects of sustainable anaesthetic care include energy use (e.g. heating, ventilation, and air conditioning systems) and circularity of medical products (e.g. reusable instead of disposable equipment).^36^

### Conclusions

We developed and pioneered the ‘Dutch Approach’ to reduce carbon emissions originating from inhalation anaesthesia. Comprehensive patient safety studies and in-depth interviews with anaesthetists were combined to create a national guideline. Meeting the two main concerns among anaesthetists, patient safety and professional autonomy, the guideline's main message is ‘TIVA when possible, inhalation anaesthesia when necessary’. Uptake of the intervention is promoted by the Dutch Society for Anaesthesiology and through inclusion in the mandatory quality control audits.

## Authors’ contributions

Study design: all authors

Conceived the idea: JMK, LG, NSW

Data extraction and analysis: JMK, LG

Drafting of the initial manuscript: JMK, EMvB, LG

Critical revisions of the manuscript: EMvB, LG, NHSW

Project supervision: NHSW, LG

Guarantors: JMK and NHSW

The corresponding author attests that all authors meet authorship criteria and that no others have been omitted.

## Funding

Dutch Society for Anaesthesiology (2023 Sustainability Grant to JMK).

## Declaration of interests

NHSW is a frequent speaker at public and private events concerning sustainability in healthcare, for which he has received travel reimbursements, but no other financial payment. NHSW chairs the Sustainability Taskforce of the Dutch Society for Anaesthesiology (NVA) and is a member of the Sustainability Committee of the European Society of Anaesthesiology and Intensive Care (ESAIC). He is a section editor (anaesthesiology and intensive care) for the Amsterdam Medical Student Journal (AMSj).
