# Supplementary material for: A nationwide approach to reduction in anaesthetic gas use: the Dutch Approach to decarbonising anaesthesia
Source: Br J Anaesth. 2025 Jan 30;134(4):1146–52. doi: 10.1016/j.bja.2024.11.049 (PMC11947575; doi:10.1016/j.bja.2024.11.049)
Supplement: Multimedia component 1 [file mmc1.docx]

**Supplementary content**

*Title*

Nationwide reduction in anaesthetic gas use: the Dutch approach to decarbonising anaesthesia

*Authors*

Jasper M Kampman, Egid M van Bree, Lieke Gielen, Nicolaas H Sperna Weiland

**Table of contents**

Appendix 1: Usage inventory questionnaire page 2

Appendix 2: Interview guide for anaesthetist interviews page 4

Appendix 3: Sustainability section in the national guideline page 6

Appendix 4: Example local protocol on anaesthetic gas use page 7

**Appendix 1: Usage inventory questionnaire distributed to every anaesthesia practice in the Netherlands (translated from Dutch to English)**

1. What type of organisation do you represent?
2. How many FTE of anaesthetists work in your organisation?
3. Can you make an assumption of the number of procedures that are performed every year in your organisation? Please separate general anaesthesia and procedural sedation, as well as adults (18 and above) and children (under 18).
4. What is the lowest fresh gas flow that anaesthetists in your practice feel comfortable with during general anaesthesia using inhalational anaesthesia?
   1. >2 litre/minute
   2. 1-2 litre/minute
   3. <1 litre/minute
   4. Not applicable
5. Can you give an indication in what percentage of cases anaesthetists in your practice use nitrous oxide as an adjuvant to general anaesthesia?
6. Can you give an indication in what percentage of cases anaesthetists in your practice use nitrous oxide as an aduvant to procedural sedation?
7. Can you give an indication what percentage of procedures under inhalational anaesthesia, total intravenous anaesthesia and nitrous oxide are performed in the following departments?
   1. Operating theatre
   2. Intensive care unit
   3. Emergency room
   4. Pediatric ward
   5. Labour ward
   6. Other
8. Does your organisation have local policy on the use of different types of anaesthetics, for instance when they can be used or when not?
   1. No
   2. Yes, based on clinical considerations
   3. Yes, based on clinical and environmental considerations
   4. Yes, …
9. Please provide for each of the following indications if it is a reason to use either inhalational anaesthesia, total intravenous anaesthesia or nitrous oxide.
   1. Preference anaesthetist
   2. Preference surgeon / other
   3. Preference patient
   4. Fast recovery / discharge
   5. Low chance of awareness
   6. Hemodynamic stability
   7. Low incidence of adverse events (e.g. PONV)
   8. Ease of use
   9. Low costs
   10. Environmental considerations
   11. Not applicable (we do not use this type of anaesthesia)
   12. Other, …
10. Do clinical considerations play a role related to certain medical disciplines or patient populations (e.g. cardiology, gynaecology, paediatrics, etc)?
11. What changes do you recognise concerning the use of different types of anaesthesia?
    1. No changes
    2. More inhalational anaesthesia compared with total intravenous anaesthesia
    3. More total intravenous anaesthesia compared with inhalational anaesthesia
    4. More nitrous oxide
    5. Less nitrous oxide
    6. Unclear whether there are changes
    7. Other, …
12. Please provide below the number of **millimetres** of inhalational anaesthesia (desflurane, isoflurane and sevoflurane) and propofol, and the number of **litres** of nitrous oxide that your organisation procured in the years 2019 to 2022.
13. What do you think are the biggest frustrations among anaesthetists when using total intravenous anaesthesia?
14. What do you think anaesthetists like best about using inhalational anaesthesia?
15. What do you think that might be reasons for anaesthetist to refrain from switching to total intravenous anaesthesia?
16. In what way can the switch from inhalational anaesthesia to total intravenous anaesthesia be made easier for anaesthetists?
17. Do you have any remaining comments concerning the questionnaire?

**Appendix 2: Interview guide for anaesthetist interviews (translated from Dutch to English)**


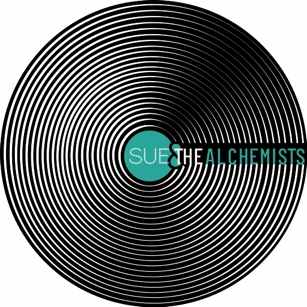


1. **Introduction**

- Can you tell me about yourself? How do your describe your work? *[how many yours of experience]*
- For which hospital do you work? *[certain types of procedures that you see more often?]*
- What makes you happy at work? When are you successful at work? What were your moments of success? *What accomplishments in the last six months are you most proud of?*
- What do you sometimes lose sleep about concerning work? What challenges do you experience? *Looking at last week, what frustrated you most?*
- What do you think are the premier challenges to an anaesthetist these days?

1. **Anaesthetics & Choice**

- What anaesthetics do you (most) use? Are there more options available that you do not use? Why not? What other anaesthetic options would you like to introduce?
- How do you make the choice? What do you think is the most important moment to decide what type of anaesthetic to use?
  - When do you use what option? *Are there other situations / contexts that influence this choice? [emergency, clinical considerations, patient specific considerations?]*
  - Who or that influences this choice? What do you see as the role of each person involved?
- What are advantages of inhalational anaesthesia? Sevoflurane? Nitrous oxide? What are disadvantages? Can you give an example?
- What are advantages of total intravenous anaesthesia? Propofol? What are disadvantages? Can you give an example?
- How would you rank the anaesthetics that you use according to environmental sustainability? Are there differences in sustainability based on use?
- Do you have a preference regarding anaesthetics for children? Why?
  - Advantages / disadvantages of inhalational and intravenous anaesthesia? Can you give an example?

1. **Behaviour – during the operation(1 per interview)**

Can you talk us through the last time that you used anaesthetic xxx>?

- What kind of patient was it? For what did he/she need anaesthesia? Was the procedure planned long in advance?
- If we go back to that day, can you take us step for step through the process, starting at coming up with the plan and ending with the patient waking up.
  - What were positive experiences? Why?
  - What were negative experiences? Why?
- What were decisive factors to not work with anaesthetic <xxx>? Why? To what degree did the procedure / team / hospital that day play a role?
- How environmentally sustainable was the procedure?
- What are positive things about this anaesthetic according to your experiences?
- Did you ever have negative experiences with this anaesthetic? What was it? Can you talk us through it? What happened?

1. **Sustainability**

- What does sustainability mean for you in your work? Can you give an example of when you paid specific attention to sustainability?
- How sustainable is anaesthesiology? Compared with other disciplines? Which discipline is doing best and who are lagging?
- If you compare anaesthesia with aviation, agriculture, the car industry, where do you stand?
- What are action / changes that you would like to see to encourage sustainability?
- What do you think are the biggest barriers for other anaesthetists to embrace the use of total intravenous anaesthesia? (projective)
- Can you list things that you think might help other anaesthetists to embrace the use of total intravenous anaesthesia?

**Optional questions**

1. **Future**

- How does the future of anaesthesiology look like? Which anaesthetic drugs should be most prominent?
- What change would you like to see in your discipline? If you could magically change one thing, what would it be? What would it improve?

1. **Authority**

- From whom would you accept advice / position in the field of anaesthesiology? From whom would you not?
- Would you like to see the development of a national guideline on the sustainable use of anaesthetics? Which organisation should develop such a guideline?

1. **Expectations of the Dutch Society for Anaesthesiology (in Dutch: NVA)**

- What do you think of the NVA? What are the first things that come to mind when you think of the NVA?
- What feeling does the NVA give you?
- Are there things that frustrate / irritate you about the NVA?
- What work of the NVA do you like? How do they help you?


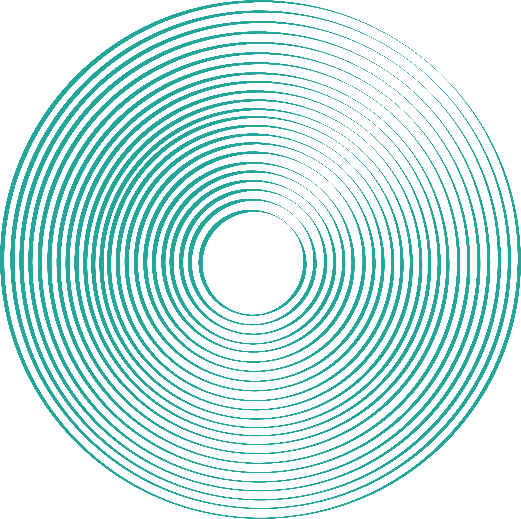

© All rights reserved by SUE & The Alchemists.

**Appendix 3: Sustainability section in the national guideline (translated from Dutch to English)**

**Sustainable anaesthesia: TIVA when possible, inhalational when necessary**

A sustainable anaesthetic practice is broader than the use of intravenous anaesthesia alone. The NVA document ’13 recommendations for anaesthesia practices to green the OR’, the ESAIC Declaration of Sustainability and the NVVH document ‘Sustainability in guidelines’ can serve as inspiration.

*Intravenous anaesthetics versus inhalational anaesthesia*

This section arises from a dilemma. Anaesthetists always put the patient on number one and use their expertise to choose the medications that are most safe and appropriate. Meanwhile, perioperative care contributes to healthcare’s burden to society and nature. An increasing number of industries face restrictive legislation. We want to steer clear of this. We want to inspire and motivate each other to reduce our healthcare-related emissions now that we have the chance. With the drive to improve our field and embrace new techniques, anaesthetists can make a considerable difference. That is why the starting point is: TIVA when possible, inhalational when necessary. This way we as anaesthetists can make a large contribution to a greener OR.

- In general, total intravenous anaesthesia (TIVA) is the first choice for general anaesthesia. When inhalational anaesthesia is clinically necessary, sevoflurane should be used.
- Indications for the use of inhalational anaesthesia should be documented, e.g. in children when establishing intravenous access is problematic.
- Minimise the environmental damage when using sevoflurane, e.g. by using end-tidal controlled dosing with ultra-low flow (<0.5 L min^-1^).
- The use of nitrous oxide should be avoided as much as possible.

The points above should be adopted and described in a local protocol.

**Appendix 4: Example local protocol on anaesthetic gas use (translated from Dutch to English)**

**Sustainable use of anaesthetics**

Protocol Department of Anaesthesiology

**Background and aim**

Volatile anaesthetics and nitrous oxide are very potent greenhouse gases^1^. Research shows that their use contributes substantially to the entire environmental footprint of surgical care^2^. By adopting a sustainable practice regarding these anaesthetics, anaesthetists can reduce the environmental impact of operations. That is why the key message is: TIVA when possible, inhalational only when necessary.

**Anaesthetics for general anaesthesia**

Total intravenous anaesthesia (TIVA) with propofol is the standard choice for induction and maintenance of general anaesthesia. For the following indications, the use of sevoflurane can be considered:

- *[Describe what applies to your practice, for example: mask induction, allergies, loss of iv access]*

**Efficient use of inhalational anaesthetics**

- There is no added benefit to using desflurane, isoflurane or nitrous oxide in anaesthesia.
- When sevoflurane is administered end-tidal controlled dosing should be used whenever possible.
- When sevoflurane is administered for the maintenance of general anaesthesia, the fresh gas flow should be kept at a minimum, preferably <0.5 L min^-1^. Research has shown that a gas flow of 0.5 L min^-1^ reduces sevoflurane use with 60% compared with a fresh gas flow of 2 L min^-13^.
- For mask induction, the system can be primed with 30 seconds of 6 L min^-1^ fresh gas flow with a sevoflurane concentration of 8%. After this, the fresh gas flow can be lowered to 1 L min^-1^ for the remainder of the induction. Research has shown that the time and quality of induction remains constant while the use of sevoflurane is reduced by 50%^4^. In some cases (e.g. when the seal to the face is inadequate), it can be necessary to increase the fresh gas flow.

**Nitrous oxide**

Research has shown that the added benefit of nitrous oxide during perioperative care is very limited, both during maintenance^5^ of induction of anaesthesia^6^.

**References**

1 Sulbaek Andersen MP, Nielsen OJ, Sherman JD. Assessing the potential climate impact of anaesthetic gases. *The Lancet Planetary Health* 2023; **7**: e622-e9

2 MacNeill AJ, Lillywhite R, Brown CJ. The impact of surgery on global climate: a carbon footprinting study of operating theatres in three health systems. *The Lancet Planetary Health* 2017; **1**: e381-e8

3 Sinnige JS, Hollmann MW, Sperna Weiland NH. Can Amsorb Plus® reduce the consumption of sevoflurane? *Canadian Journal of Anaesthesia/Journal canadien d'anesthésie* 2022; **69**: 274-5

4 Singh A, Sinha R, Aravindan A, Kumar KR, Datta PK. Comparison of low-fresh gas flow technique to standard technique of sevoflurane induction in children—A randomized controlled trial. *Pediatric Anesthesia* 2019; **29**: 304-9

5 Kampman JM, Plasmans KYQ, Hermanides J, Hollmann MW, Repping S, Sperna Weiland NH. Influence of nitrous oxide added to general anaesthesia on postoperative mortality and morbidity: a systematic review and meta-analysis. *British Journal of Anaesthesia* 2024

6 Gordon DW, Chatterjee D, McGain F. It's time to stop using nitrous oxide for pediatric mask induction. *Pediatric Anesthesia* 2024; **34**: 104-7
